# Exploring 97 Years of Aedes aegypti as the Vector for Dengue, Yellow Fever, Zika, and Chikungunya (Diptera: Culicidae): Scientometric Analysis

**DOI:** 10.2196/65844

**Published:** 2025-04-23

**Authors:** Yoon Ling Cheong, Sumarni Mohd Ghazali, Mohd Hazilas Mat Hashim, Mohd Khairuddin Che Ibrahim, Afzufira Amran, Tsye Yih Tiunh, Hui Li Lim, Yong Kang Cheah, Balvinder Singh Gill, Kuang Hock Lim

**Affiliations:** 1 Biomedical Museum Unit, Special Resource Centre Institute for Medical Research, National Institutes of Health Ministry of Health Malaysia Kuala Lumpur Malaysia; 2 Biomedical Epidemiology Unit, Special Resource Centre Institute for Medical Research National Institutes of Health, Ministry of Health Malaysia Selangor Malaysia; 3 Biomedical Research, Strategic & Innovation Management Unit Institute for Medical Research, Director's Office National Institutes of Health, Ministry of Health Malaysia Selangor Malaysia; 4 Clinical Research Centre National Institutes of Health Ministry of Health Malaysia Selangor Malaysia; 5 School of Economics, Finance & Banking Universiti Utara Malaysia Kedah Malaysia; 6 Special Resource Centre Institute for Medical Research, National Institutes of Health Ministry of Health Malaysia Selangor Malaysia

**Keywords:** relative growth rate, vector control, collaboration index, bibliometric, Aedes aegypti, Scopus, co-occurrence, author, dengue, Zika

## Abstract

**Background:**

*Aedes aegypti* is an important vector that transmits dengue, Zika, chikungunya, and yellow fever viruses. Although research on *Aedes aegypti* has been conducted for decades, scientometric studies on *Aedes aegypti* are scarce, are limited to regions, and cover short periods. Thus, there is still a knowledge gap in the current trend, research focuses and directions, leading authors and collaboration, journal and citation impacts, countries, and worldwide collaborations.

**Objective:**

The objectives of the study are to investigate the research trend, focus and directions, citation impact, leading authors and collaboration, journals, and countries of the published works on *Aedes aegypti* to inform the current knowledge gaps and future direction of the control of the vector.

**Methods:**

In this study, we searched the Scopus database for articles on *Aedes aegypti* published from the year 1927 until April 5th, 2024, and included articles, reviews, books, and book chapters that were written in English. A total of 16,247 articles in 160 journals with 481,479 citations were included. Inconsistencies in authors’ names were checked and cleaned using OpenRefine. The data were grouped into 4 periods; years 1927-1999, 2000-2009, 2010-2019, and 2020-2023. The relative growth rate and doubling time of publications were calculated. The analysis was conducted using VOSviewer, R bibliometrics, and citeSpace.

**Results:**

The overall RGR was 0.1. Doubling time increased from 9.3 in 1978-1998 to 12.1 in 2000-2009. The main research clusters were “using Wolbachia,” “Dengue Zika,” “worldwide diversity,” “community support,” “larvicidal activity,” “mosquito genotype-dependent,” and “sterile insect technique.” *Journal of Medical Entomology* was the leading journal (758/16,247, 4.7%). The most cited articles were authored by Halstead SB and team in *Science* (N=1355) and Kraemer MU and team in *eLife* (N=1324). The United States (5806/23,538, 24.7%) and Brazil (2035/23,538, 8.6%) were the top countries. Gubler DJ was the top co-cited author (n=2892) from 2000 to 2019. The co-cited author cluster patterns informed the significant specialty research on *Aedes aegypti* across time. Authors from various specialized research fields tended to collaborate across countries, especially neighboring countries. Countries with more research funding on the study of *Aedes aegypti* published more papers.

**Conclusions:**

Researchers or entomologists could understand the current knowledge gap on *Aedes aegypti* and plan for future research pathways. This study contributed to the public health stakeholders in improving the vector control interventions and elucidated the extent of research subject areas.

## Introduction

*Aedes aegypti* is a vector of several arboviruses, including dengue, Zika, chikungunya, and yellow fever. This species was discovered by Fredrik Hasselqvist in Egypt in 1757 [1]. Initially designated as Culex aegypti, the name was declared invalid by the International Commission on Zoological Nomenclature in 1956 and it was subsequently identified as Stegomyia fasciata followed by *Aedes aegypti* (Linnaeus) or *Aedes* (Stegomyia) *aegypti* (Linnaeus) [1]. Its distinguishing features are the presence of white rings around the leg articulations and abdomen and a white lyre-shaped marking on the dorsal surface of the thorax [2]. Originally native to sub-Saharan Africa, *Aedes aegypti* is now widespread in most tropical and subtropical locations across the world [3]. The most common habitats of the *Aedes aegypti* are artificial and natural containers, including plastic containers, flower pots, vases, tires, tree holes, and bromeliad plants [4,5]. They are common in high-density residential areas [3]. *Aedes aegypti* can potentially survive and establish in 215 of 250 countries and territories (86%) [6].

Scientometrics concerns the analysis of subjects and the development of research literature, the impact and process of scientific knowledge production, as well as the macroenvironment of research [7]. The aim is to discover hidden relationships between any single publication and citations [8]. Studies on animal species using the scientometric method revealed trends in the direction of research on animal species. Santos and Vianna [9], who conducted a scientometric analysis of the literature on 11 Western Atlantic species of Paralichthys in ISI Web of Science and SciELO, discovered the dominant fishery species of *Paralichthys dentatus* (46.1%) in Canada and *Paralichthys lethostigma* (32.1%) in the United States. Araújo [10] discovered the average number of total citations per paper was 16.05 (27.66) in the scientific literature on insect galls and galling species in Brazil from 1988 to 2017. Miguel, Calvão, Vital, and Juen [11], who examined the literature on insects of the order Odonata, found the majority of the study focused primarily on the adult stage and species level, and there were gaps in the biogeography, parasitism, competition within and across species, evolutionary and phylogenetic links, and studies of the larval stages.

To date, there is a lack of scientometric analysis of *Aedes aegypti* at the global level. Vega-Almeida et al [12] conducted a scientometric analysis of 5039 published articles about *Aedes aegypti* from the year 2006 to 2015 (10 years) in Scopus. The findings were distributed across 4 major domains, namely epidemiology, gene expression and biological control, larvicidal and insecticidal effects, and reproduction and insecticide resistance; however, the paper did not analyze the information on authorship, citation rates, journal, organization, and collaborations. Other studies focused solely on the diseases transmitted by *Aedes aegypti* [13]*,* or the effects of climate change on *Aedes aegypti* [14]. Gupta and Tiwari [15] discovered from the 910 articles publications of dengue research in India for the duration of the year 2003 to 2012, with an average annual growth rate of 28.19%. A scientometric analysis of the Zika virus, yielded 567 publications, with the most productive countries being the United States, the United Kingdom, and the Netherlands [16]. Sindhania et al [17] focused on the publication on *Aedes aegypti* and *Aedes albopictus* extracted from the Web of Science, encompassing a collection of 4149 papers for 77 years. Focus on single species was crucial as the targeted vector controls of both species were differed due to the uniqueness of their preferable habitats, morphology, genetic profile, and virus replication characteristics [18,19]. Hence, a scientometric study on *Aedes aegypti* based on Scopus that covers a wider aspect, including author collaborations and cocitations, citation rate and impact, leading journals, and countries would enable researchers and entomologists to map the current knowledge gap on *Aedes aegypti* and to plan future research focus or priorities. The Scopus offers more multidiscipline contents than Web of Science [20]. For public health practitioners, this information would assist in improving vector control interventions and evaluating the extent of past and potential research areas.

This study aims to conduct a scientometric analysis of scientific articles on *Aedes aegypti* published worldwide from 1927 to 2023. The specific objectives are numerous; first, to analyze the general trends and annual growth rate of the articles published on *Aedes aegypti*; second, to identify prolific authors, their collaborative networks, and authorship patterns; third, to determine the co-occurrences, patterns of subject areas, and current research trends; fourth, to study the journals and their citation impact; and finally, to determine the countries of the published papers.

## Methods

### Data Source and Search Strategy

We conducted our search in the Scopus database for publications from the year 1927 to 2023 using the search term “Aedes aegypti” on April 15, 2024 (Textbox 1). The following search queries were used in the investigation in Scopus in two phases. First, search all the publications up to the year 2023.

Second, search the publications by 4 time periods, that was years 1927-1999, 2000-2009, 2010-2019, and 2020-2023.

(TITLE-ABS-KEY(“Aedes aegypti”) AND PUBYEAR > 1926 AND PUBYEAR < 2024 AND ( LIMIT-TO ( DOCTYPE,“ar” ) OR LIMIT-TO ( DOCTYPE,“re” ) OR LIMIT-TO ( DOCTYPE,“ch” ) OR LIMIT-TO ( DOCTYPE,“bk” ) ) AND ( LIMIT-TO ( LANGUAGE,“English” ) ) ).

The search was conducted within the title, abstract, and keywords fields and was limited to articles, reviews, books, and book chapters in English. Documents that did not match the inclusion criteria were excluded. The excluded articles included retracted articles, conference papers, letters, notes, editorials, short surveys, erratum, conference reviews, and data reviews. Data extracted from the search results included publication year, title, author, citation, keywords, organization, journal, and country, and were saved in 5 “csv” format files. Inconsistencies in authors’ names were checked and cleaned using OpenRefine [21]. For quality control, a random sample of 10% of the extracted data was verified against the original source publication. Data merging, pivoting, and aggregation analysis were conducted with Microsoft Excel.

### Data Analysis

#### Relative Growth Rate and Doubling Time

We calculated the relative growth rate (RGR) and doubling time of publications. RGR, which measures the change in the number of publications per year, was calculated using the following formula:



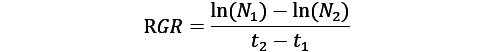



where, *t_1_* is the initial time period, *t_2_* is the final time period, *N_1_* denotes the number of publications at time *t_1_*, and *N_2_* denotes the number of publications at time *t_2_*.

Given the RGR, we computed the doubling time. Doubling time is the time it takes for the number of publications to double.



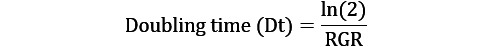



#### Authorship Analysis

Analysis of authorship and subject area and construction of the respective network graphs were conducted using VOSviewer software developed by Leiden University [22]. The coauthorship analysis was based on the full counting method, that was, each coauthor of a publication is assigned a weight of one; thereby, the total weight of a publication is equal to the number of its authors. Author contribution timelines, 3-field plots, and country collaboration maps were generated using the R bibliometrics package [23].

#### Impact Factor and Cocitation Analysis

Journal publications’ 5-year impact factor was extracted from Web of Science Journal Information. Cocitation analysis tracks pairs of study that are cited together and suggest similarity as both items were cited by the same study, which helps in identifying the inner structure of research disciplines that represent the research focus [24]. The analysis was conducted using the VOSviewer and citeSpace [25]. The author’s cocitation analysis was split into 4 time periods: years 1927-1999, 2000-2009, 2010-2019, and 2020-2023. To understand the recent trend of research focus, the citeSpace analysis zoomed in on the year 2021-2023. The higher silhouette score of a cluster means members in the cluster have more in common in terms of tightness and separation [26].

The current study focused solely on *Aedes aegypti*, with *Aedes albopictus* being the next target of investigation. This approach is due to the species-specific nature of biological control and the differing habitats of both species. Conducting a scientometric analysis on a single species, provides a clearer picture, which can later be expanded to compare trends between the 2 species.

### Ethical Considerations

Ethical approval protocol from the Medical Research and Ethics Committee, Ministry of Health Malaysia was exempted (reference number KKM/NIH/22-01333-RCQ[1]).

## Results

### General Trends

**Figure 1:**
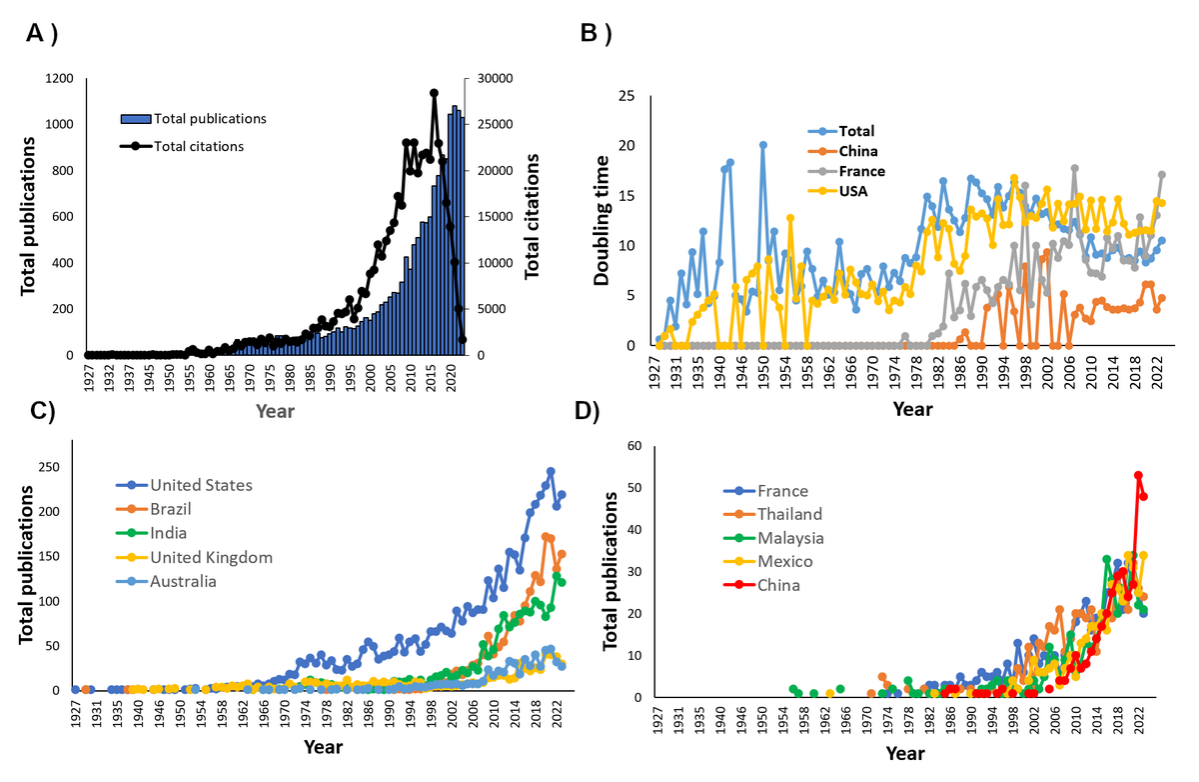
Distribution by year of (A) total number of publications and citations on Aedes aegypti globally, (B) total and selected countries’ publication doubling time, (C) total number of publications by the top 5 countries, and (D) total number of publications by the 6th-10th ranked countries.

The search strategy produced a total of 18,529 results, mainly in English (17,438/18,686, 93.3%), Spanish (506/18,686, 2.7%), Portuguese (278/18,686, 1.5%), French (203/18,686, 1.1%), and the other languages. For the articles in English (n=17,438), the document types were research articles (14,990/17,438, 86.0%), followed by review articles (1063/17,438, 6.1%), conference proceedings (366/17,438, 2.1%), letters (253/17,438, 1.5%), notes (191/17,438, 1.1%), and others. Finally, only research articles, reviews, letters, editorials, books, and book chapters were included (n=16,247). Figure 1A shows that articles were published at a steeply increasing trend with a total number of 14,990 (14,990/16,247, 92.26%). The review articles increased steadily and exhibited a doubling in number after the year 2014, contributing to a total number of 1063 papers (1063/16,247, 6.54%). There were 189 (189/16,247, 1.16%) book chapters and 5 (5/16,247, 0.03%) books on *Aedes aegypti*. The total number of articles in each of the time periods was 3183, 2506, 6345, and 4214, respectively. The number of authors expanded rapidly from 4490, 6459, and 19,495 to 18,064, respectively. The top countries published scientific articles on “Aedes aegypti*”* were the United States (5806/23,538, 24.7%), followed by Brazil (2035/23,538, 8.6%), India (1839/23,538, 7.8%), United Kingdom (1243/23,538, 5.3%), France (898/23,538, 3.8%), and Australia (779/23,538, 3.3%; Figure 1C). The other countries with a significant number of articles were Thailand (678/23,538, 2.8%), Mexico (548/23,538, 2.3%), China (541/23,538, 2.3%), and Malaysia (455/23,538, 1.9%), respectively (Figure 1D).

The overall RGR was 0.1. Doubling time increased from 9.3 in 1978-1998 to 12.1 in 2000-2009, and reverted back to 9.3 in 2020-2023 (Figure 1B). The RGR and DT varied across countries. Among the top 10 countries in total publications, France exhibited the longest doubling time, while China had the shortest doubling time. The Unites States has published since 1927 and had a short doubling time initially, but the numbers reduced in recent years (Figure 1B).

### Institutional Contributions

Authors affiliated with the Fundacao Oswaldo Cruz produced 3.4% of the overall *Aedes aegypti* research output, followed by those in the University of Florida (2.3%), CNRS Centre National de la Recherche Scientifique (1.8%), Institut Pasteur, Paris (1.7%), Colorado State University (1.6%), Mahidol University (1.6%), Centers for Disease Control and Prevention (1.5%), University of Notre Dame (1.5%), Universidade de São Paulo (1.5%), and University of California, Davis (1.5%).

### Funding Agencies

There were 159 funding agencies for the *Aedes aegypti* research whilst 8914/19,883 (50.2%) articles did not disclose their sources of funding. The National Institute of Allergy and Infectious Diseases, United States (1716/19,883; 9.7%) and National Institutes of Health, United States (1183/19,883; 6.67%) contributed to a quarter of the publications. Four agencies sponsored more than 200 articles each, that was, Conselho Nacional de Desenvolvimento Científico e Tecnológico, Brazil (708/19,883, 4.0%); Coordenação de Aperfeiçoamento de Pessoal de Nível Superior, Brazil (576/19,883, 3.2%); National Science Foundation, United States (255/19,883, 1.4%); and Centers for Disease Control and Prevention, United States (255/19,883, 1.4%). For other regions and countries, the highest funders were National Natural Science Foundation of China (207/19,883, 1.2%); Wellcome Trust (158/19,883, 0.9%); National Health and Medical Research Council, Australia (149/19,883, 0.8%); Horizon 2020 Framework Programme, European Union (144/19,883, 0.8%); Department of Science and Technology, Ministry of Science and Technology, India (108/19,883, 0.6%); Natural Sciences and Engineering Research Council of Canada (78/19,883, 0.4%); Consejo Nacional de Ciencia y Tecnología, Mexico (77/19,883, 0.4%); and Agence Nationale de la Recherche, France (84/19,883, 0.5%).

### Authorship Analysis

The top 20 productive authors who published articles related to *Aedes aegypti* originated from mixed countries, mainly the United States, for that were, Scott TW, Raikhel AS, Severson DW, Christensen BM, James AA, Becnel JJ, Tabanca N, Ali A, and Higgs S (Table 1). Three authors from Australia were among the first 10 productive authors, including Ritchie SA (n=126), Hoffmann AA (n=94), and O’Neill SL (n=79). The other authors were from France (Failloux A-B, n=95), Thailand (Chareonviriyaphap T, n=78), Italy (Benelli G, n=74), India (Govindarajan M, n=69), Malaysia (Lee HL, n=64), Mexico (Manrique-Saide P, n=63), Brazil (LourenÇo-de-oliveira R, n=55), and Trinidad and Tobago (Chadee DD, n=55). The author with the highest number of publications did not have the highest citation and H-index. The highest H-index author was Hoffmann AA (H-index=103) and the author with the highest total citation was Scott TW (n=14,100).

**Table 1:** Top 20 productive authors, citation, and Scopus H-Index.

Authors	Country	Total publication, n (%)	Total citation, n (%)	Scopus H-index (2024)
Ritchie SA	Australia	126 (0.78)	7522 (1.56)	59
Scott TW	United States	116 (0.71)	14,100 (2.93)	82
Raikhel AS	United States	115 (0.71)	9074 (1.88)	68
Severson DW	United States	108 (0.66)	5756 (1.20)	44
Failloux A-B	France	95 (0.58)	5370 (1.12)	53
Hoffmann AA	Australia	94 (0.58)	5659 (1.18)	103
Christensen BM	United States	88 (0.54)	3923 (0.81)	49
O’Neill SL	Australia	79 (0.49)	8050 (1.67)	77
Chareonnviriyaphap T	Thailand	78 (0.48)	1807 (0.38)	37
James AA	United States	77 (0.47)	5618 (1.17)	59
Benelli G	Italy	74 (0.46)	5394 (1.12)	84
Becnel JJ	United States	74 (0.46)	2026 (0.42)	43
Govindarajan M	India	69 (0.42)	3440 (0.71)	59
Tabanca N	United States	66 (0.41)	1545 (0.32)	38
Lee HL	Malaysia	64 (0.39)	1618 (0.34)	30
Manrique-Saide P	Mexico	63 (0.39)	1161 (0.24)	24
Ali A	United States	57 (0.35)	1232 (0.26)	22
Lourenço-de-Oliveira R	Brazil	55 (0.34)	4396 (0.91)	49
Chadee DD	Trinidad and Tobago	55 (0.34)	1855 (0.39)	35
Higgs S	United States	54 (0.33)	5149 (1.07)	67

**Figure 2:**
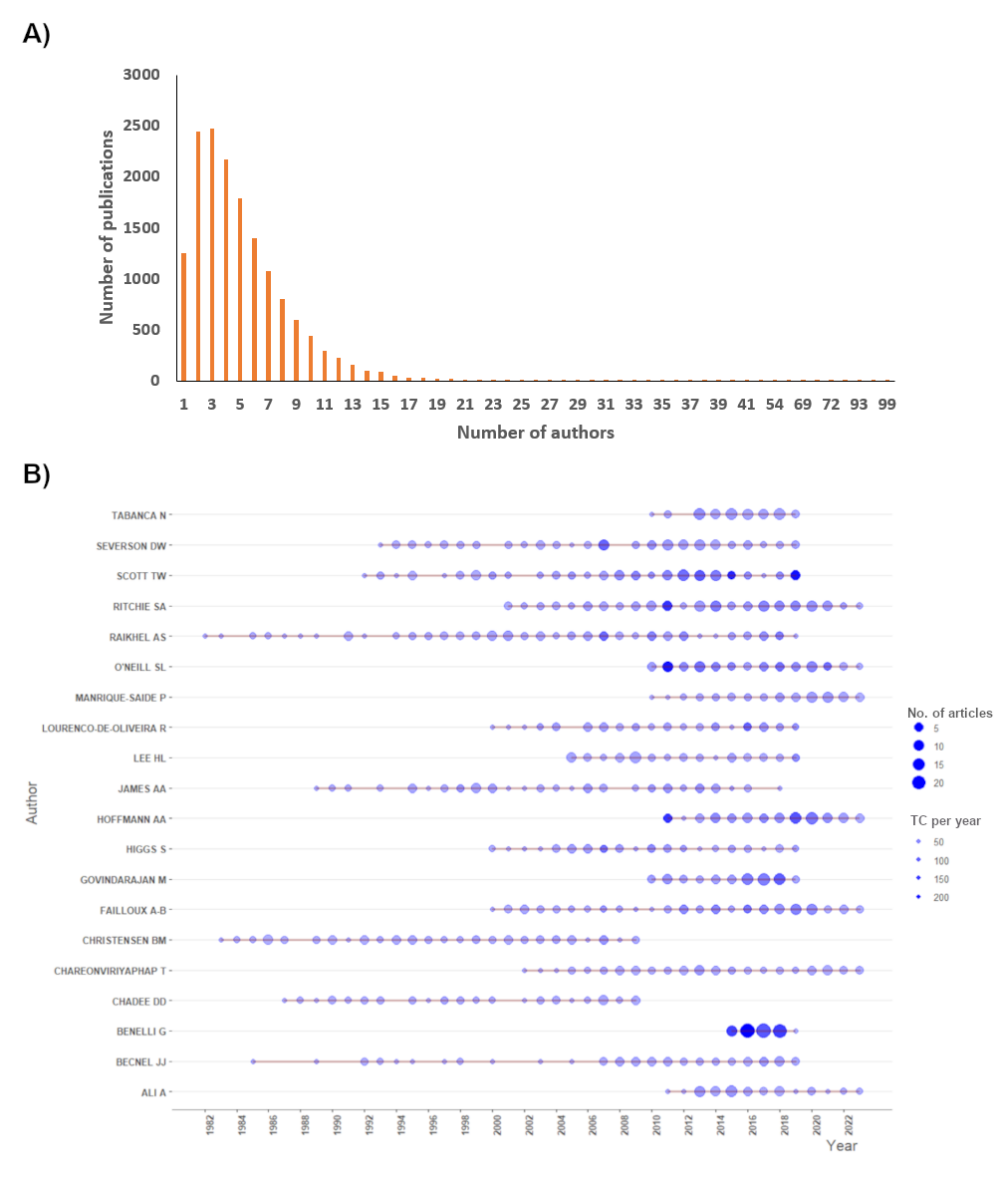
Author analysis. (A) Number of publications by number of authors, and (b) top 20 authors’ production over time and total citations per year.

The majority of the publications had between 2 and 5 authors. The number of publications decreased exponentially with increases in number of coauthors. There were more publications by multiple authors than by single authors (Figure 2A). The top-ranked authors were Raikhel AS, Christensen BM, Becnel JJ, and Chadee, each of whom has published articles on *Aedes aegypti* since 1982 (Figure 2B). Benelli G published several articles with high citations in a span of 4 years. Hoffmann AA, Manrique-saide P, Ritchie SA, O’Neill SL, Failloux AB, Chareonviriyaphap T, and Ali A are still actively publishing study on *Aedes aegypti* (up to 2023; Figure 2B).

**Figure 3:**
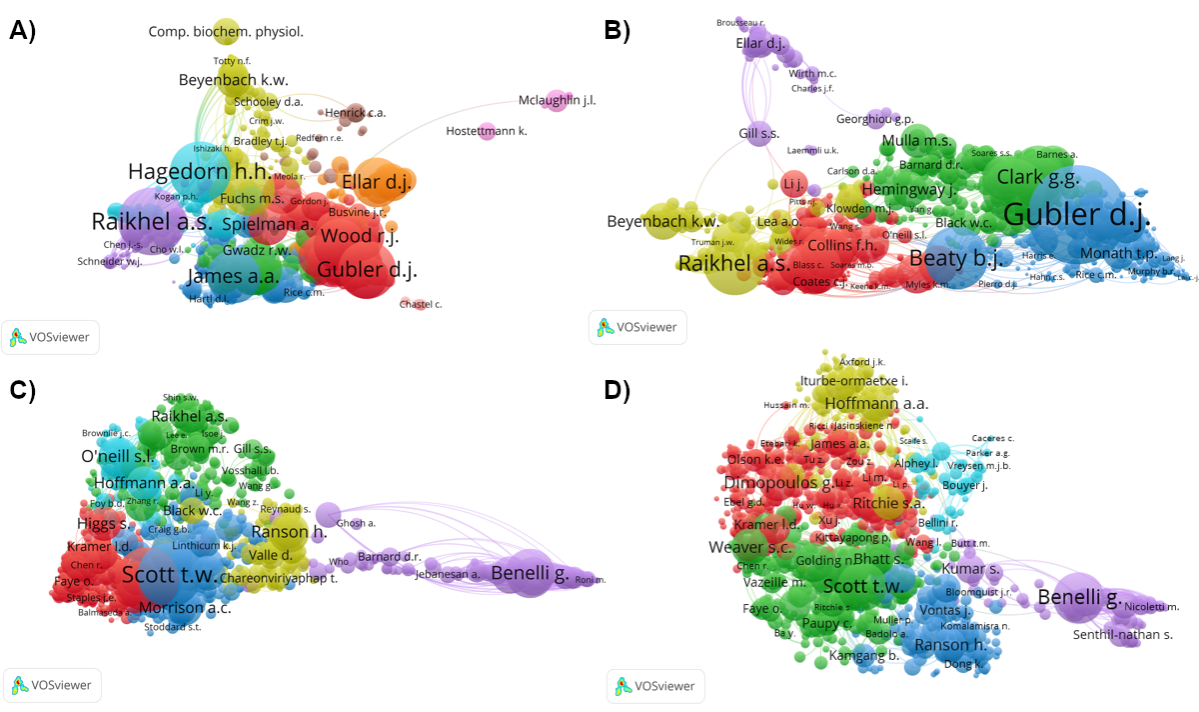
Cocitations network of authorship for the period of (A) 1927-1999, (B) 2000-2009, (C) 2010-2019, and (D) 2020-2023.

In the early years, of all the 8975 cited authors, Raikhel AS had the highest number of cocitation links with other authors (n=303), followed by Hagedorn HH (n=248) and James AA (n=204; Figure 3A). From 2000 through 2009, of all the 71,005 cited authors, Gubler DJ was the most cocited author (n=1272), followed by Beaty BJ (n=711) and Raikhel AS (Figure 3B). From 2010-2019, Gubler DJ remained as the top co-cited author (n=2892) among 219,499 cited authors. Scott TW was the second co-cited author (n=2885), while Beneli G was the third (n=1986; Figure 3C). From 2020 to 2023, out of 230,547 cited authors, Scott TW (n=1311) and Hoffmann AA (n=858) stayed the highest in the cocitation list, with Brady OJ (n=1086) came in third (Figure 3D). The varied color depicted authors’ linkages with different clusters. Gubler DJ, Brady OJ, and Scott TW were in the same cluster, whereas Hoffmann AA and Ritchie SA were in another cluster.

**Figure 4:**
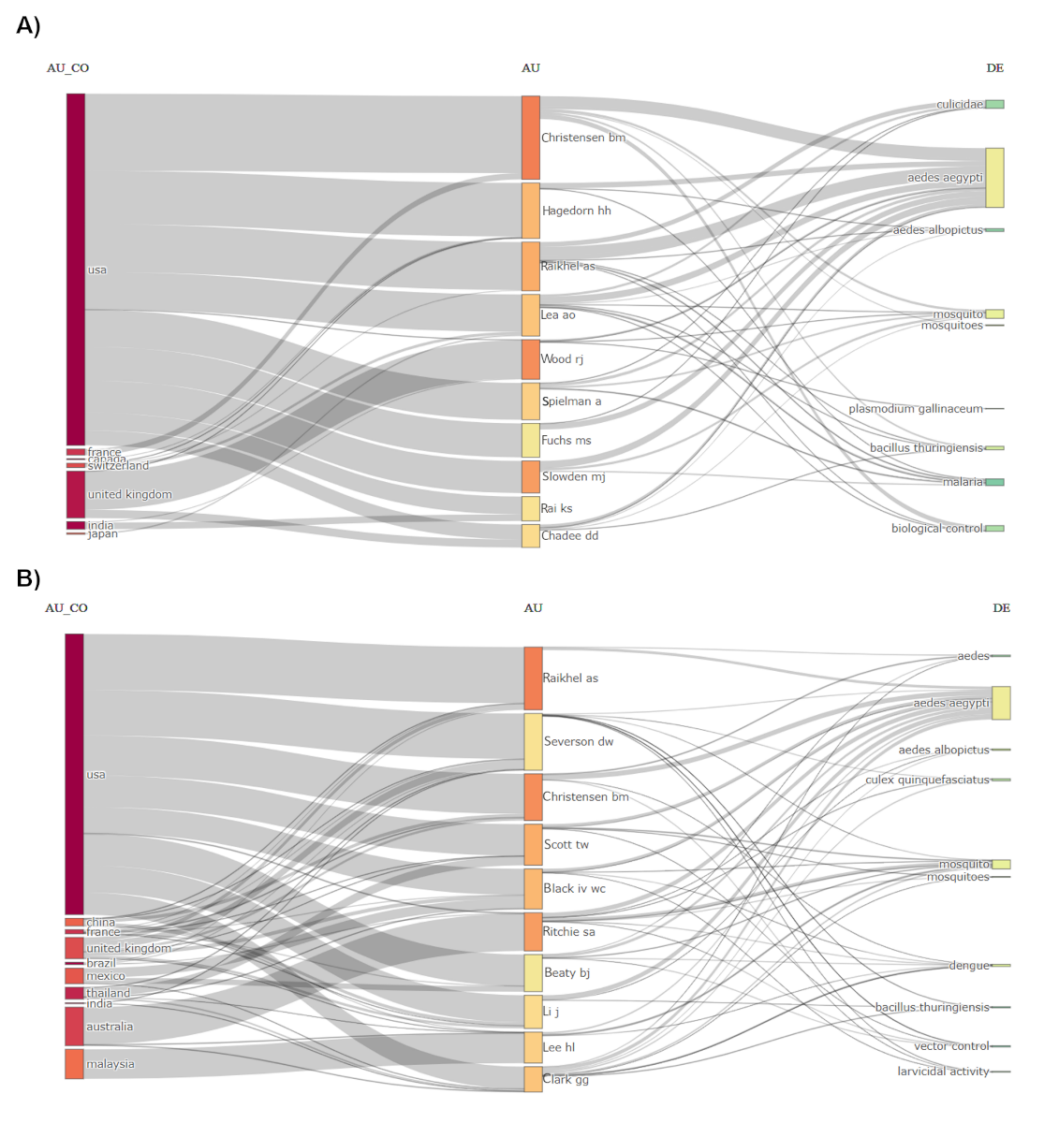
Three-field plot (Sankey diagram) of country, author, and keywords of the cited references for the 10 most productive authors from (A) 1927 to 1999 and (B) 2000 to 2009. AU: authors; AU_CO: countries; DE: keywords.

**Figure 5:**
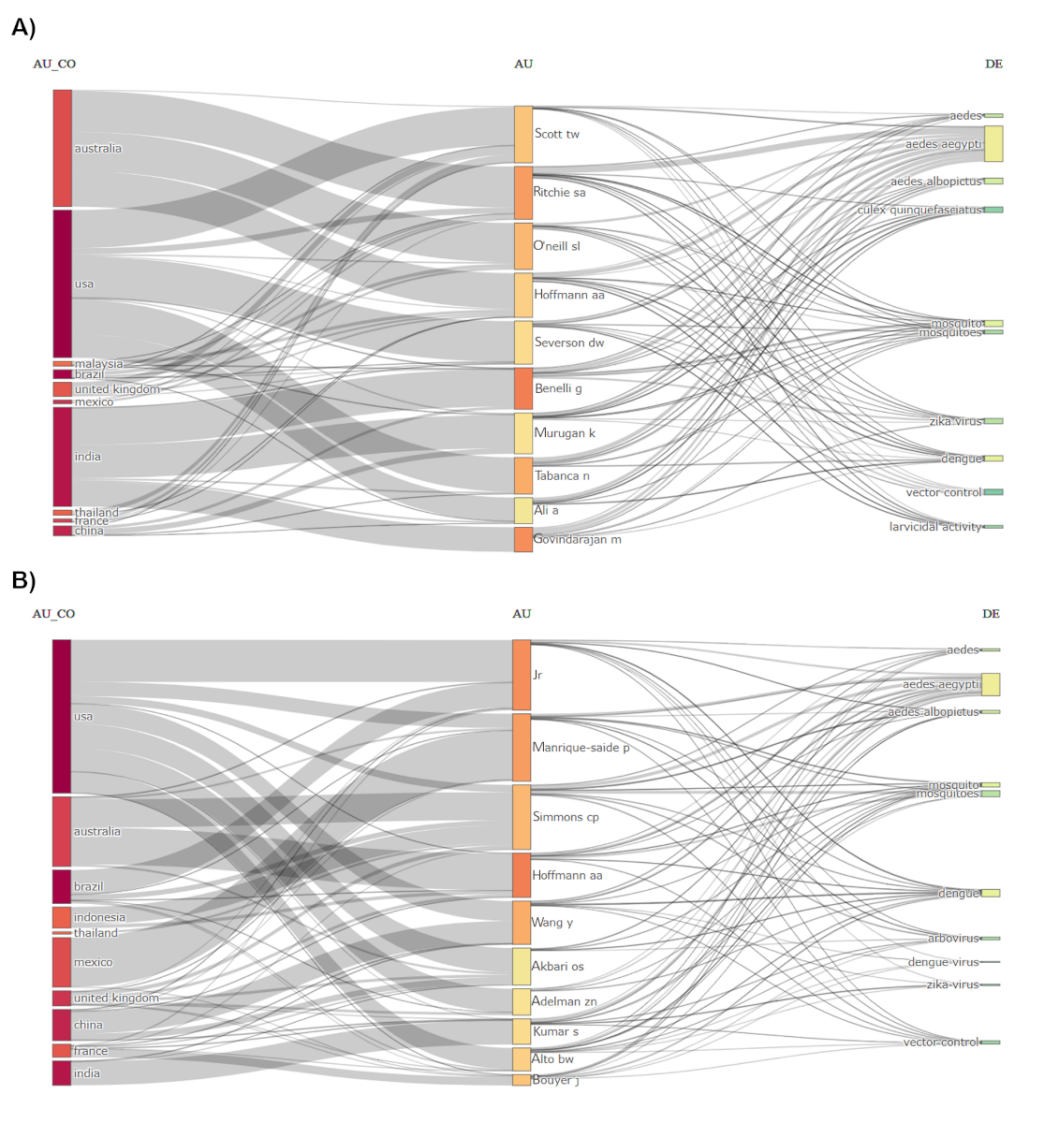
Three-field plot (Sankey diagram) of country, author, and keywords of the cited references for the 10 most productive authors from (A) 2010 to 2019 and (B) 2020 to 2023. AU: authors; AU_CO: countries; DE: keywords.

Figure 4 depicts the country of origin of the top authors and their research keywords for each period. In the initial period from 1927-1999, about 90% of the top 10 authors were affiliated with the United States and focused on the study of the Culicidae, *Aedes albopictus*, *Plasmodium gallinaceum*, *Bacillus thuringiensis*, malaria, and biological control (Figure 4A). From 2000 to 2009, more authors from other countries published on *Aedes aegypti*, that was Ritchie SA from Australia and Lee HL from Malaysia. The focus of research has shifted from Malaria to dengue and the additional species that was included was *Culex quinquefasciatus* (Figure 4B). The larvicidal activity was also the focus of the research. From 2010 to 2019, three main authors from Australia topped the productive list, namely Ritchie SA, O’neil SL, and Hoffmann AA, while authors from India contributed many articles, including Benelli G, Murugan K, and Govindarajan M. The top authors focused not only on dengue but also Zika virus (Figure 5A). In the recent period (2020-2023), authors from the United States, Australia, Mexico, China, Brazil, and India were equally productive in publishing their research on *Aedes aegypti* and on similar keywords as the previous periods (Figure 5B).

### Subject Area and Research Trends

Most of the publications were in journals within the subject areas of medicine (7860/30,051; 26.2%); agricultural and biological sciences (5863/30,051; 19.5%); immunology and microbiology (5150/30,051; 17.1%); biochemistry, genetics, and molecular biology (4078/30,051; 13.6%); veterinary science (1513/30,051; 5.0%), and environmental science (1194/30,051; 4.0%). From 2000 to 2019, physics- and astronomy-related publications exhibited the highest growth rate (84), followed by engineering (9.7) and mathematics (8.54).

**Figure 6:**
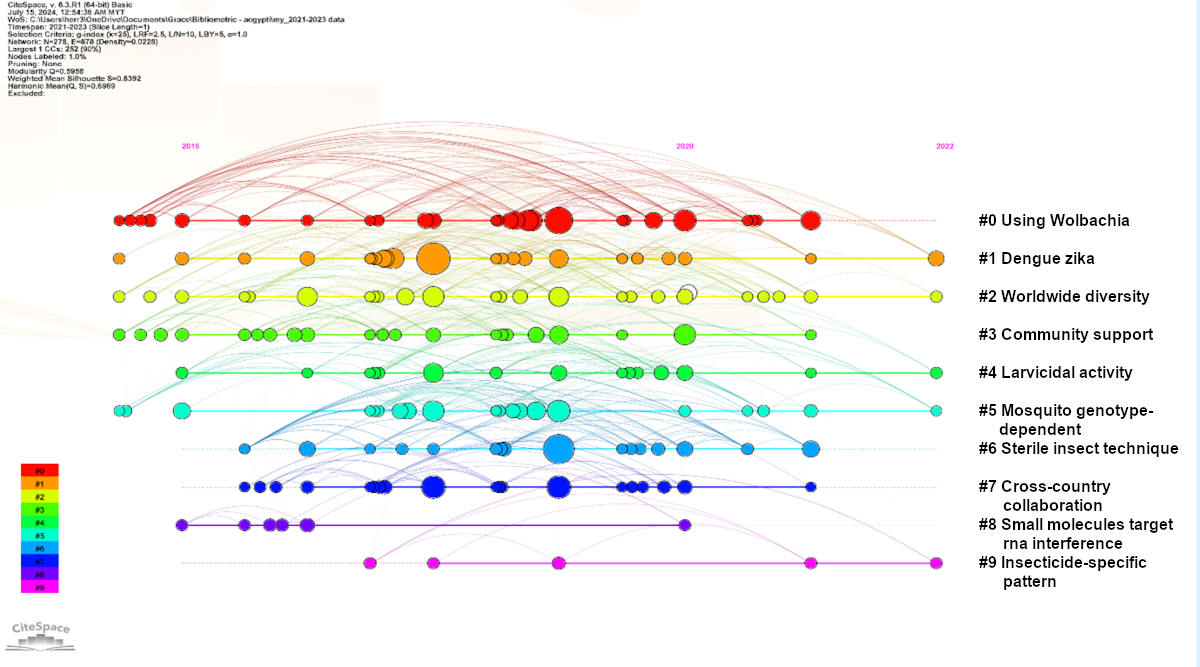
Visualization of the reference cocitation network time map (Year 2021-2023).

We identified 31 research clusters based on the cocitation reference network. From 2021 to 2023, the temporal evolution of the research topic clusters was discovered with the indication of the cluster silhouette score, size, and mean year of co-cited papers. The research clusters were categorized into 5 major research trends, that was, biological vector control, chemical vector control, and genetic of *Aedes aegypti*, disease-related and collaboration efforts. The main cluster, biological vector control, referred to the largest cluster, #0, concerning “using Wolbachia” (score=0.793; size=42; year=2018) [27] and cluster #6 on “sterile insect technique (SIT)” (score=0.856; size=25; year=2019) [28] (Figure 6). SIT started gaining attention after Wolbachia (#0), as one of the biological vector controls using sterilized male *Aedes aegypti* with either irradiation or exposure to a chemical sterilant in damaging somatic tissue to reduce the mosquito population [29]. The second major research trend concerning disease-related focused on “dengue Zika,” #1, (score=0.839; size=35; year=2019) [19]. The third major research trend was related to collaboration efforts, which referred to “worldwide diversity,” #2, (score=0.814; size=33; year=2018) [30], “community support,” #3, (Score=0.834; size=29; year=2017) [31] and “cross-country collaboration,” #7, (score=0.8; size=24; year=2018) [32]. The research related to chemical vector control contributed to the fourth research trend: “larvicidal activity,” #4, (score=0.883; size=26; year=2018) [33] and “insecticide-specific pattern,” #9, (score=0.971; size=6; year=2019) [34]. The fifth research trend was related to genetics: “mosquito genotype-dependent,” #5, (score=0.877; size=26; year=2018) [35] and “small molecules target RNA interference,” #8, (score=0.932; size=6; year=2017) [36].

There were other significant subject areas from the analysis of keywords co-occurrences across 4 periods. Before the year 2000, the focused subject areas included *Bacillus thuringiensis*, electron microscopy, chromosome mapping, virus cultivation, and mosquito control (Figure S1 in Multimedia Appendix 1). From year 2000 to 2009, the research subject area related to *Aedes aegypti* focused on Culicidae, molecular sequence data, insecticides, bacterial toxins, larvae, insecticides, and dengue virus (Figure S2 in Multimedia Appendix 1). The subject areas were then extended to insecticidal activity, insect repellent, bioassay, plant extract, metabolism, genetics, insect proteins, physiology, disease transmission, and population density from the year 2010 to 2020 (Figure S3 in Multimedia Appendix 1). In the recent 4 years from 2020 to 2023, the subject areas focused on insecticide resistance, temefos, pyrethrins, larvicidal activity, metabolism, gene expression, reverse transcription polymerase, prevalence, vector control, arbovirus infections, and Zika virus (Figure S4 in Multimedia Appendix 1). The research focuses on *Aedes aegypti* linking with dengue stayed relatively important throughout the period. The number of publications focused on yellow fever were low and linked to *Aedes aegypti* solely (Figures S5-S8 in Multimedia Appendix 1). The research on chikungunya were not closely related to others research topics in the early period and were only started linking with dengue in the period of 2010-2019 and connecting with Zika in the period of 2020-2023. In 2010-2019, Zika started to become closely related topic with dengue and chikungunya in addition later on.

### Citation Analysis

The top 10 papers with the highest number of citations are listed in Table 2. The top cited article was “Pathogenesis of dengue: challenges to molecular biology” authored by Halstead SB [37] (n=1355). The second highest cited article was “The global distribution of the arbovirus vectors Aedes aegypti and Aedes albopictus,” authored by Kraemer et al [18] (n=1324), followed by the “A Wolbachia symbiont in Aedes aegypti limits infection with dengue, chikungunya, and Plasmodium. Cell. 2009” by Moreira et al [38] (n=1196).

**Table 2:** Top 10 cited papers.

Article authors and title	Journal	Year	Citation, n (%)	Average citations per year
Halstead SB. Pathogenesis of dengue: challenges to molecular biology [37].	*Science*	1988	1355 (0.28)	38.7
Kraemer MU, Sinka ME, Duda KA, Mylne AQ, Shearer FM, Barker CM, Moore CG, Carvalho RG, Coelho GE, Van Bortel W, Hendrickx G. The global distribution of the arbovirus vectors Aedes aegypti and Aedes albopictus [18].	*eLife*	2015	1324 (0.27)	165.5
Moreira LA, Iturbe-Ormaetxe I, Jeffery JA, Lu G, Pyke AT, Hedges LM, Rocha BC, Hall-Mendelin S, Day A, Riegler M, Hugo LE. A Wolbachia symbiont in Aedes aegypti limits infection with dengue, Chikungunya, and Plasmodium [38].	*Cell*	2009	1196 (0.25)	85.4
Gubler DJ. Epidemic dengue and dengue hemorrhagic fever as a public health, social and economic problem in the 21st century [39].	*Trends in Microbiology*	2002	1191 (0.25)	56.7
Dudchenko O, Batra SS, Omer AD, Nyquist SK, Hoeger M, Durand NC, Shamim MS, Machol I, Lander ES, Aiden AP, Aiden EL. De novo assembly of the Aedes aegypti genome using Hi-C yields chromosome-length scaffolds [40].	*Science*	2017	1136 (0.24)	38.7
Tsetsarkin KA, Vanlandingham DL, McGee CE, Higgs S. A single mutation in Chikungunya virus affects vector specificity and epidemic potential [41].	*PLoS Pathogens*	2007	1127 (0.23)	70.4
Musso D, Gubler DJ. Zika virus [42].	*Clinical Microbiology Reviews*	2016	1112 (0.23)	158.9
Weaver SC, Reisen WK. Present and future arboviral threats [43].	*Antiviral Research*	2010	1060 (0.22)	38.7
Hoffmann AA, Montgomery BL, Popovici J, Iturbe-Ormaetxe I, Johnson PH, Muzzi F, Greenfield M, Durkan M, Leong YS, Dong Y, Cook H. Successful establishment of Wolbachia in Aedes populations to suppress dengue transmission [44].	*Nature*	2011	1052 (0.22)	87.7
Cugola FR, Fernandes IR, Russo FB, Freitas BC, Dias JL, Guimarães KP, Benazzato C, Almeida N, Pignatari GC, Romero S, Polonio CM. The Brazilian Zika virus strain causes birth defects in experimental models [45].	*Nature*	2016	1003 (0.21)	143.3

### Journals

The total number of journals that published papers on *Aedes aegypti* from 1928 to 2023 were 160. The leading journal that published articles on the *Aedes aegypti* were *Journal of Medical Entomology* (JME; 758/16,247, 4.7%), *PLOS* *Neglected Tropical Diseases* (NTD) (604/16,247, 3.7%), *Journal of the* *American Mosquito Control Association* (AMCA) (505/16,247, 3.1%), *Parasites and Vectors* (493/16,247, 3.0%), and *PLOS One* (401/16,247, 2.5%; Table 3). The journal with the highest number of citations for their publication on *Aedes aegypti* was PLOS NTD (n=25,274), followed by JME (n=23,682) and the *American Journal of Tropical Medicine and Hygiene* (AJTMH; n=19,281; Table 3).

**Figure 7:**
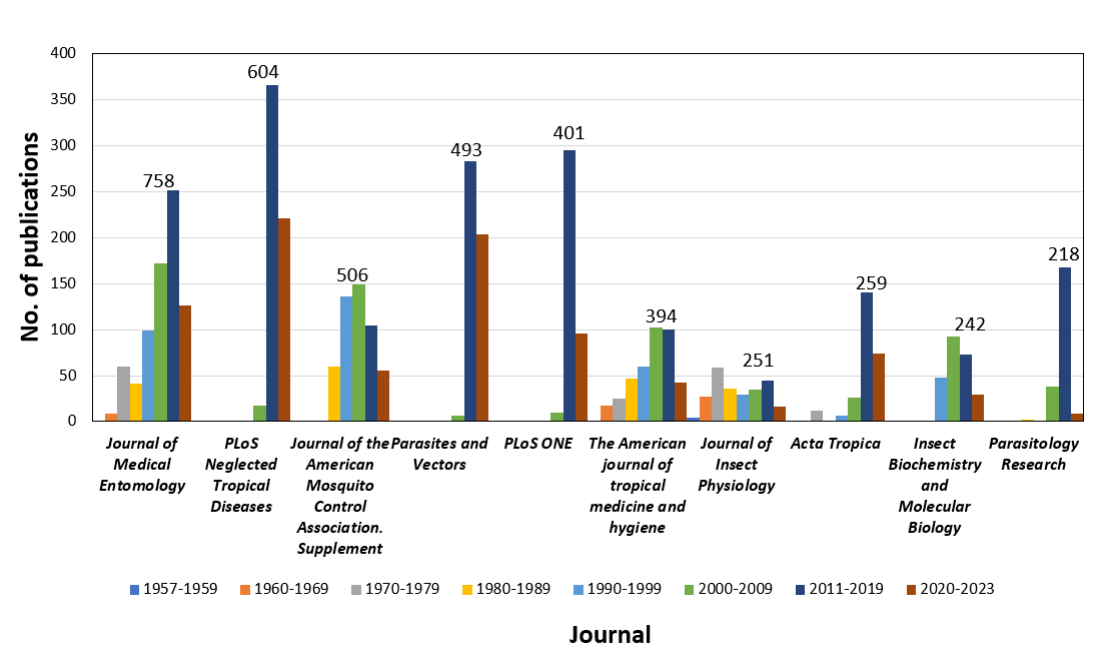
Number of publications by the top 10 journals by decade.

Figure 7 shows the distribution of publications on *Aedes aegypti* by journal across the years from 1957 to 2023. The *Journal of Insect Physiology* was the earliest to publish on *Aedes aegypti,* that was, since 1957, whereas the AJTMH and JME began publishing on *Aedes aegypti* between 1960 and 1970. From 2011 to 2019, most study were published in *PLOS NTD*, *Parasites and Vectors*, *PLOS One*, *Parasitology Research*, and *Acta Tropica*.

**Table 3:** Top 10 journals by number of papers on Aedes aegypti (from 1927-2023) and 5-year impact factor (2019-2023).

Journal	Number of papers (N=16,247), n (%)	Total citations (N=481,479), n (%)	5-year impact factor in 2023
*Journal of Medical Entomology*	758 (4.67)	23,682 (4.92)	2
*PLOS* ^a^ *neglected tropical diseases*	604 (3.72)	25,274 (5.25)	3.6
*Journal of the American Mosquito Control Association*	505 (3.11)	10,818 (2.25)	1
*Parasites and Vectors*	493 (3.03)	11,896 (2.47)	3.3
*PLOS One*	401 (2.47)	14,877 (3.09)	3.3
*American Journal of Tropical Medicine and Hygiene*	357 (2.2)	19,281 (4.00)	2.2
*Acta Tropica*	259 (1.59)	6312 (1.31)	2.4
*Journal of Insect Physiology*	251 (1.54)	8193 (1.70)	2.3
*Insect Biochemistry and Molecular Biology*	242 (1.49)	11,658 (2.42)	3.7
*Parasitology Research*	218 (1.34)	11,767 (2.44)	1.9

### Country Analysis

**Figure 8:**
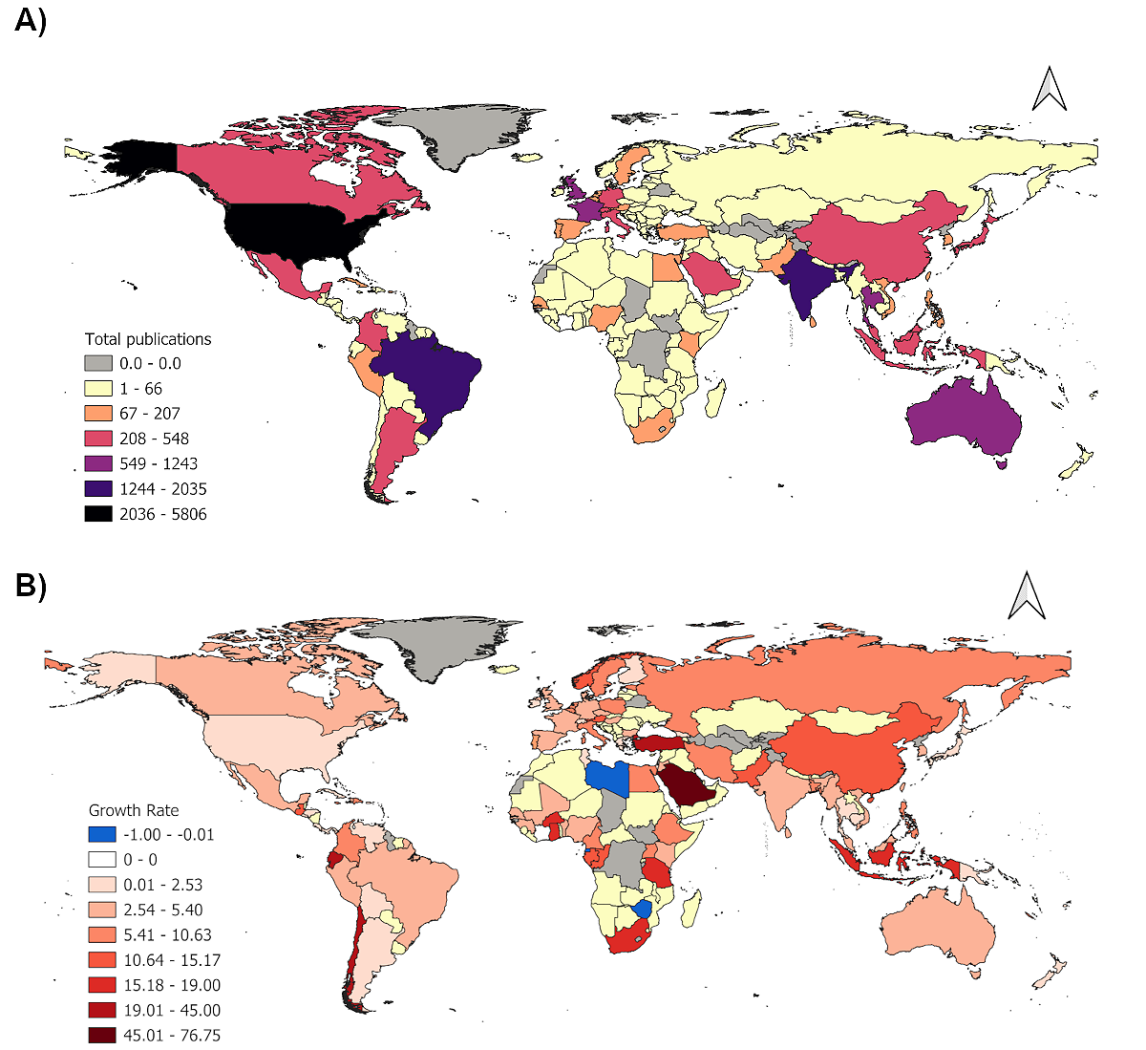
A map of the (A) total publications of Aedes aegypti by country for the years 1927-2023 and (B) growth rate of the scientific articles on Aedes aegypti by country for the year 2000-2009 to 2010-2023.

More than one-third of the publications during the study period were by authors from the United States (5806/23,538, 24.7%), Brazil (2035/23,538, 8.6%), and India (1839/23,538, 7.8%). The next top 7 countries were from Europe (United Kingdom: 1243/23,538, 5.3%; and France: 898/23,538, 3.8%), Asia (Thailand: 678/23,538, 2.9%; Malaysia: 455/23,538, 1.9%; and China: 541/23,538, 2.3%), Mexico (548/23,538, 2.3%), and Australia (779/23,538, 3.3%). The decadal growth rate (from 2000 to 2019) of the articles by country was topped by Saudi Arabia (76.75), followed by Turkey (45), Chile (29), Ecuador (24), and Ghana (19) (Figure 8A). High decadal growth rates were also observed for Africa (Burkina Faso: 17.67; South Africa: 16.33; and the United Republic of Tanzania: 16), Asia (Hong Kong: 18; Indonesia: 16.6; and Pakistan: 14.5), Europe (Austria: 15.17; Norway: 13.5; and Estonia: 9), South America (Colombia: 9.52; French Guiana: 5.4; and Brazil: 4.82), Australia (4.17), and the United States (2.46; Figure 8B). 

**Figure 9:**
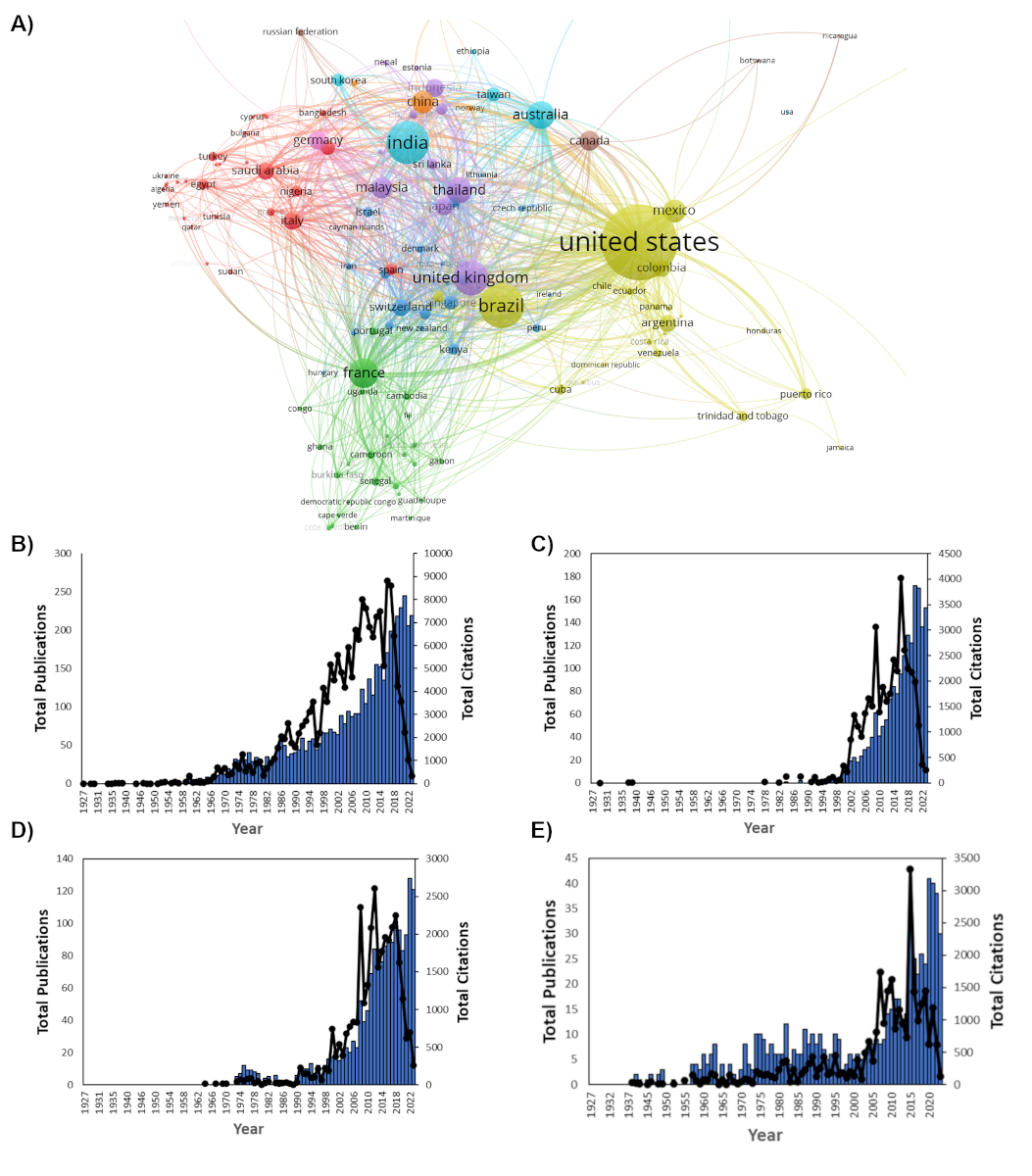
Co-authorship analysis of 131 countries. (A) Co-authorship by country: publications and total citations in the top 4 countries including (B) the United States, (C) Brazil, (D) India, and (E) the United Kingdom.

Coauthorship analysis of 131 countries revealed that the majority of coauthors were authors from the United States who collaborated with authors from Brazil and South America (Figure 9). Authors from India collaborated with Australian, Taiwanese, and South Korean authors. UK authors mainly collaborated with Thai, Malaysian, and Sri Lankan authors. Authors in France collaborated primarily with countries in Africa including Uganda, Congo, Cameron, Senegal, and Ghana. The European authors, that was, in Germany, Italy, and Spain worked closely with the collaborators from Saudi Arabia, Bangladesh, Egypt, and Turkey.

The country with the highest total citations was the United States (182,414/481,479, 37.89%), which was 5 times higher than the second highest country, Brazil (39,115/481,479, 8.12%). Switzerland (7469/481,479, 1.55%) and Italy (7,239/481,479, 1.50%) were both in the top 10 highest number of citations, even though they had low numbers of articles (Switzerland: n=302; and Italy: n=307). The rest of the countries in the top 10 list of total citation based on origin of corresponding author were India (32,646/481,479, 6.78%), United Kingdom (30,328/481,479, 6.30%), Australia (25,242/481,479, 5.24%), France (23,626/481,479, 4.91%), Thailand (12,154/481,479, 2.52%), and Mexico (6450/481,479, 1.34%).

## Discussion

### Principal Findings

The medically important mosquitoes, in particular, *Aedes aegypti*, has been studied for centuries. Female *Aedes aegypti* was one of the main vectors for transmitting viruses, including dengue virus, Zika virus, chikungunya virus, and yellow fever virus. In this study, the detailed evaluation of the *Aedes aegypti* published works informed the current knowledge gaps and future direction of the control of the vector. During the study period, 16,247 items were published and cited 481,479 times. In the recent 3 years, the literature focused on 5 areas: biological vector control studies, chemical vector control studies, genetic studies, disease-related studies, and collaboration efforts. The minor changes in the research trend were observed when comparing Vega-Almeida et al [12] (year 2006-2015) with the research focus on epidemiology, gene expression and biological control, larvicidal and insecticidal effects, and reproduction and insecticide resistance. The biological vector control using Wolbachia has been gaining more attention as many countries proved its efficiency in reducing dengue cases [44,46,47]. The ongoing Sterile insect techniques pilot projects in many tropical and sub-tropical countries have shown the effectiveness of using irradiated mosquitoes in reducing the wild population [48]. The research direction will be the biological vector control projects at a larger scale or national operational level in the future. Lately, dengue, Zika, and chikungunya were emerging research topics related to *Aedes aegypti*. In recent years, there has been a noticeable rise in the number of articles addressing *Aedes aegypti* in discussing the effectiveness of vector control strategies. One of the control measures of the effectiveness of vector control strategies was the insecticide resistance of *Aedes aegypti*. Insecticides remain widely used in vector control interventions resulted in mosquito resistance to insecticides in 4 classes, including organophosphates, carbamates, pyrethroids and the organochlorine dichlorodiphenyltrichloroethane [49], and more in recent years. The insecticide resistance level of *Aedes aegypti* were conducted with various methods, for example, the WHO (World Health Organization) tube test [50], WHO bottle bioassay [51-53], the Centers for Disease Control and Prevention’s bottle bioassay [54,55]. The WHO showed capital interest in the insecticide resistance study on malaria and dengue vector mosquitoes that several guidelines were provided to discuss the monitoring of insecticide resistance in mosquito vectors [56,57].

Genetic studies in relation to the gene knockdown of insecticides and the genomic profiles and relationship of the vector and patient were also the main research focus in recent years as the knowledge of genes requires more exploration and elucidation. The detailed genome mapping, RNA-seq data alignment, and gene expression quantification methods were investigated in detail to understand the relationship between mosquito genotype and the microbes in the science of vector mosquito genetic control [35,58,59]. Zika is still without cure and the symptoms could be severe, such as Guillain-Barré syndrome, congenital malformations in infancy, neuropathy, and myelitis in adults and older children. Many current studies focused on the vector competence of *Aedes aegypti*, particularly the Zika virus strain’ infectivity, dissemination, and transmission rates as well as the influence of the larval microbiome on mosquito genotype-dependent [35,60]. Understanding the antiviral pathway in using the small molecule RNA interference and jak or stat signaling is crucial in controlling Zika virus infection in *Aedes aegypti* [36,61].

The booms of publications on *Aedes aegypti* after the year 2000 may be due to the increase in dengue cases globally, from 505,430 to 6.5 million from the year 2000 to 2023 [62,63]. Since 2007, there have been several Zika outbreaks in Africa, the Americas, Asia, and the Pacific. Zika was briefly declared as a Public Health Emergency of International Concern in 2016 [64]. The threat of chikungunya outbreaks in recent decades was unprecedented, especially in 2013-14 in the Caribbean and Latin America [65]. Moreover, the WHO declared the yellow fever as a global epidemic threat due to international travel [65]. The number of publications on *Aedes aegypti* and other vector is expected to increase in the future if infectious diseases caused by this vector remain uncontrolled or are exacerbated by climate change, rapid urbanization, and international human mobility. In the recent 2 decades, there was a surge of the growth rate of the publication on *Aedes aegypti*. The dengue endemic countries in the region of Western Asia, South America, and Western and Southern Africa produced more research output focus on the *Aedes aegypti*. However, countries in the northern and central Africa have been underrepresented in the research studies on *Aedes aegypti*. Studies in Burkina Faso, Ghana, South Africa, and Tanzania-countries with higher growth rate in publications on *Aedes aegypti*–can serve as the valuable references for other African nations with similar human environment in the study of origin, biology, behaviors, habitat, insecticide sensitivity of vectors, etc [66-70].

The analysis of coauthorship revealed that publications on *Aedes aegypti* were primarily collaborative efforts involving multiple authors from various specialized research fields. Authors from the United States constituted 45% of the top 20 most productive authors. This was likely due to increased access to funding resources. The top 20 productive authors originated from Australia, Asia (Thailand, India, and Malaysia), South America (Brazil and Trinidad and Tobago), and Europe (France and Italy). Authors who published on diseases transmitted by *Aedes aegypti* that are of global relevance, such as Gubler DJ and Halstead SB, had high numbers of citations and co-citations. During the Zika outbreak, authors who published on *Aedes aegypti* received more citations because of the increased public health interest in disease patterns, transmission links, and vector control methods related to Zika [42]. Often, authors gained more citations after years of experience in disseminating research findings [71].

The co-citation analysis provided a deeper understanding of vital research trends on *Aedes aegypti*. Gubler DJ was the highest co-cited author but was not among the top 20 productive authors. Gubler (H-index 94) was central in bridging researchers in the field for his significant works on dengue, antibody-dependent enhancement, flavivirus, Zika virus, microcephaly, and yellow fever. Gubler’s review papers on dengue and dengue hemorrhagic fevers globally and locally were cited by the majority of dengue research papers, especially in the introduction sections [72]. The top co-cited article also highlighted a global study similar to Gubler DJ whereas Bhatt et al (2013) mapped and discussed the global distribution of the burden of dengue. Co-citation analysis can answer the research question, “Who are the central, peripheral, and bridging researchers in the field, and how has the structure developed over time?” [73] by connecting journals, authors, and various documents [74] regarding the intellective structure. The cocitation cluster patterns provide a new way to study the specialty structure of science [75]. Further studies on the cocitation trends by the years can provide insight and understanding of how the research developed over time.

There appeared to be links between authorship, citation impacts, collaborations, and funding. Higher funding support is strongly associated with higher citation impacts [76]. Funders were acknowledged in only 52.7% of the articles, based on the available information that showed the United States contribute to most of the top cited authors and articles on *Aedes aegypti*. The United States together with Brazil published the most study on *Aedes aegypti* aligning with other worldwide dengue bibliometric studies, showing these countries were the most productive countries [77], given that *Aedes aegypti* is the main vector for dengue disease in the countries. Authors from neighboring countries tended to collaborate more within their regions. International coauthorship has a strong positive effect on the number of articles and citations a country produces [78,79]. This regional clustering is evident in Figure 8A, where the United States collaborated with Mexico, Colombia, Panama, and Argentina, while India worked with Sri Lanka, Thailand, and Bangladesh. Recently, China has shown a steep increase in publications on *Aedes aegypti* due to increased research funding and higher research focus on infectious disease [80-82].

Journal analysis provided information on the journals with the highest impact articles related to *Aedes aegypti* worldwide. The top journal, JME, a bimonthly publication focused on medical entomology and medical acarology, especially arthropods of public health importance, has published articles on *Aedes aegypti* since 1957 and exhibited an increasing trend in the number of articles for the past 60 years. JME’s impact factor was 2 (The year 2019-2023) and was the second highest in total citations. Although PLOS NTD contributed the second highest number of publications on *Aedes aegypti*, it obtained the highest number of citations since its first publication on *Aedes aegypti* in 2008 and with a recent impact factor of 3.6 (for the year 2019-2023), indicating it has a great impact. PLOS NTD was also reported as the leading journal in publications on Leishmania [83] and neglected tropical diseases [84] in the Latin Americas and the Caribbean. However, these journals were grouped into different clusters as JME published more articles on *Aedes aegypti* physiology and vector control, whereas PLOS NTD articles covered more virus and genetic studies. The 10 leading journals mostly fell in the categories of agricultural and biological sciences, immunology and microbiology, and medicine of Clarivate’s Journal Citation Report. Most of the journals that fell into multiple subject categories gained higher citations than those in single or dual categories [85].

The limitation of the current study should not be omitted. The study only sourced the data from Scopus due to the difficulties in combining the control measure across multiple databases, hence it may not capture all the papers from other databases such as Web of Science, Google Scholar, and Microsoft Academic. Besides, the qualitative components of the research were inaccessible in terms of peer review, ethical concerns and societal influence. Outputs from other academic discipline such as patents, produced systems developed and widely used, policy papers, white papers, reports produced for government and other public organizations, and exhibition were not included. In short, this study remains as valuable resources for comprehending the direction and significance of research.

### Conclusions

In conclusion, the research on *Aedes aegypti* increased at a relative growth rate of 12.1 especially in the year 2000 to 2009, with a total number of 16,247 articles in 160 journals and were cited for 481,479 times. The prolific authors were mainly from the United States and the top co-cited authors across the years were elucidated. The coauthorship was collaborative involving multiple authors from various specialized research fields and countries, especially neighboring countries. Central and bridging researchers of the study on *Aedes aegypti* were identified and discussed based on the co-citation analysis, and this will provide better understanding how the research changed over time. The researchers’ next area of interest may be the research direction. The leading journal were JME, PLOS NTD, AMCA, *Parasites and Vectors*, and *PLOS One*, with the top cited article titled “Pathogenesis of dengue: challenges to molecular biology” by Halstead SB in *Science*. Leading countries in published articles such as the United States and Brazil were also countries that funded higher number of published articles on *Aedes aegypti*.

The strengths of the study include the capability to identify the recent 3 years’ research trends: “using Wolbachia,” “Dengue Zika,” “worldwide diversity,” “community support,” “larvicidal activity,” “mosquito genotype-dependent,” “sterile insect technique,” “cross-country collaboration,” “small molecules target RNA interference,” and “insecticide-specific pattern.” The recent research mainly focused on biological control, that is, an alternative to chemical control. This will be awareness for the scientific communities on the need to study the medicinal important vector, in which the researchers or entomologists could understand the current knowledge gap on *Aedes aegypti* and to plan for future research pathways. This study also contributed to the public health stakeholder and funder on the current research direction and knowledge gap for better decision-making and research priorities in determining the suitable intervention for vector control.

## Data Availability

The datasets generated or analyzed during this study are available from the corresponding author on reasonable request.

## Appendix

Multimedia Appendix 1 Co-occurrence network of keywords.

## Abbreviations

AJTMH: American Journal of Tropical Medicine and Hygiene
AMCA: American Mosquito Control Association
JME: Journal of Medical Entomology
NTD: Neglected Tropical Diseases
RGR: relative growth rate
WHO: World Health Organization
