# Supplementary material for: Exploring 97 Years of Aedes aegypti as the Vector for Dengue, Yellow Fever, Zika, and Chikungunya (Diptera: Culicidae): Scientometric Analysis
Source: Interact J Med Res. 2025 Apr 23;14:e65844. doi: 10.2196/65844 (PMC12059494; doi:10.2196/65844)
Supplement: Multimedia Appendix 1 [file ijmr_v14i1e65844_app1.docx]

**Multimedia Appendix 1**


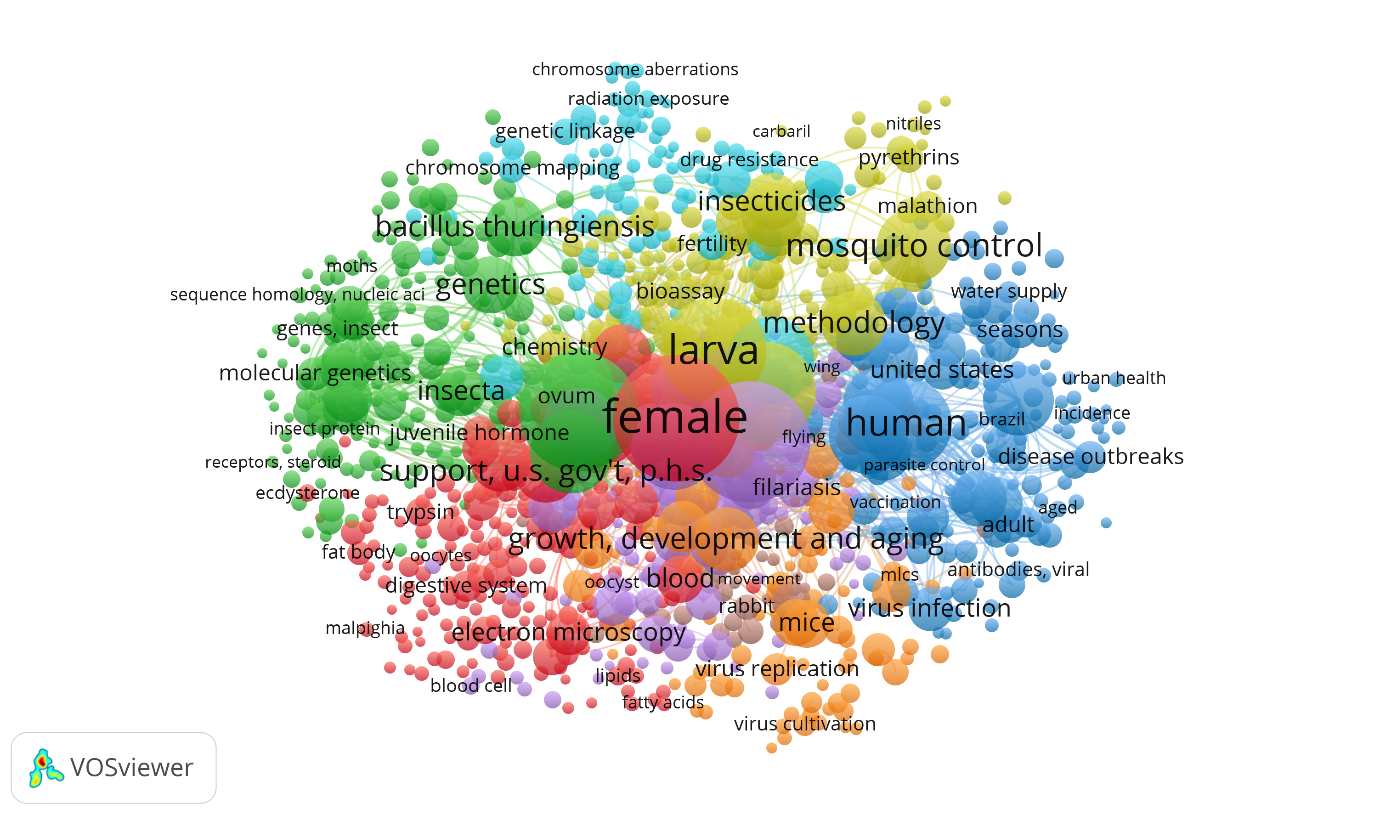


Figure S1. Co-occurrence network of keywords from 1927 to 1999.


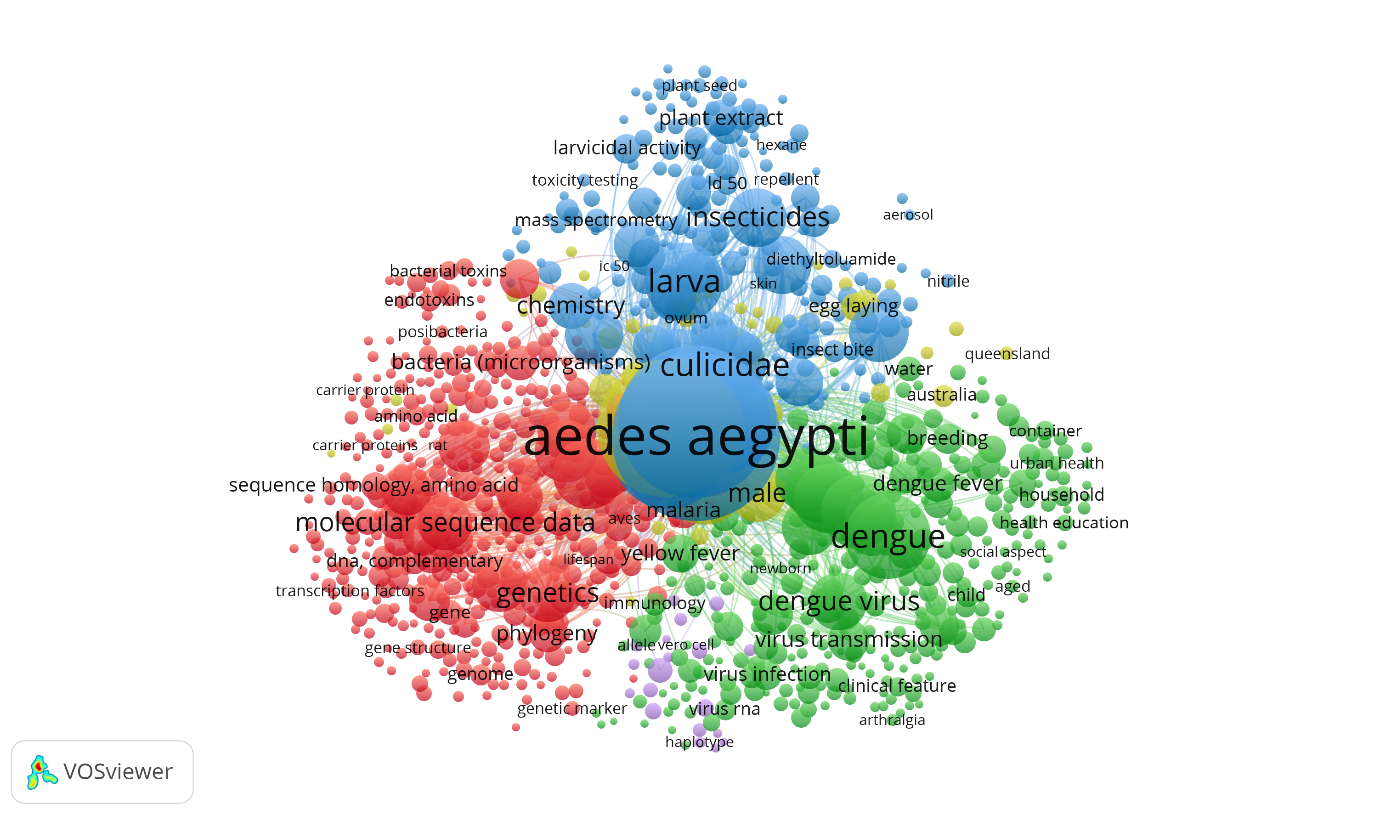


Figure S2. Co-occurrence network of keywords from 2000 to 2009.


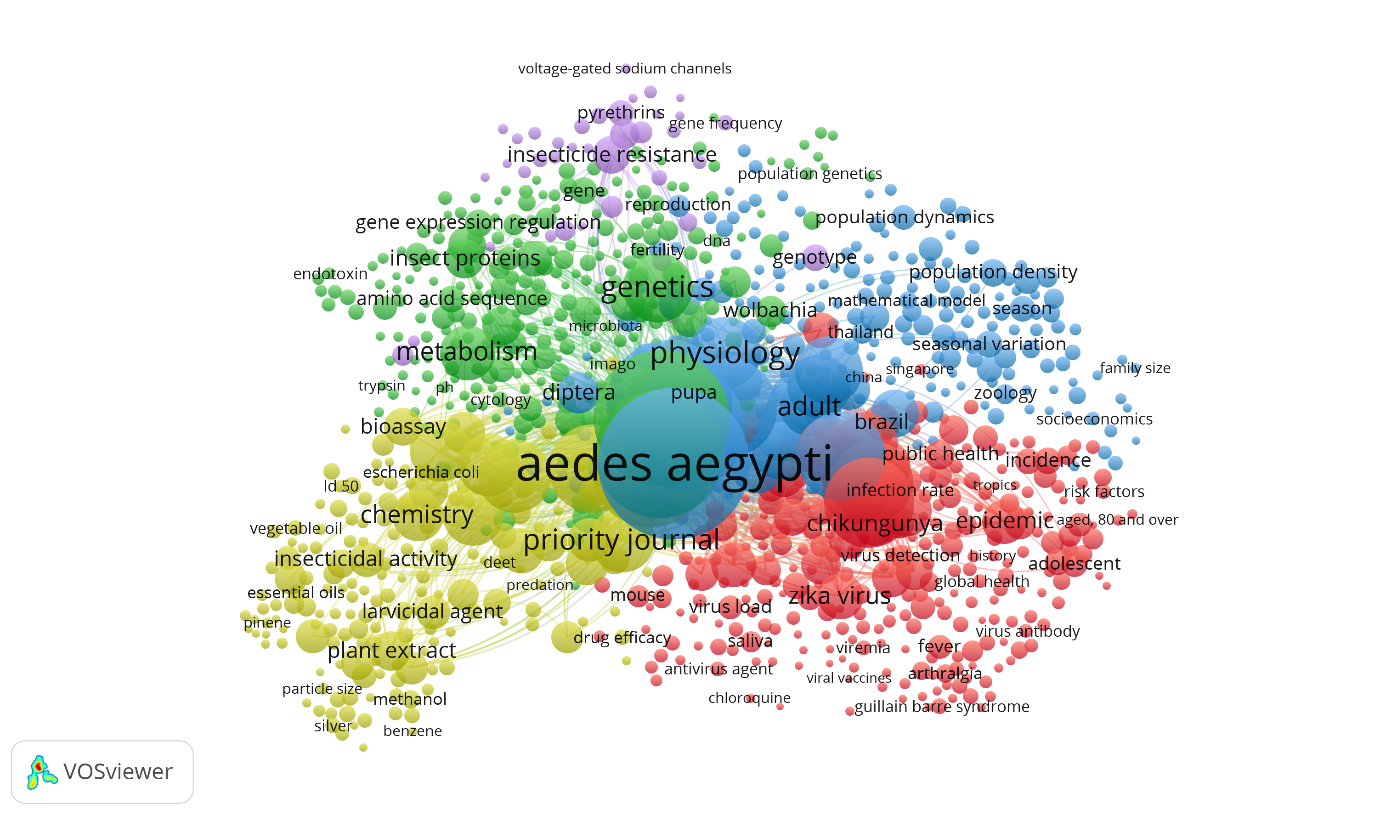


Figure S3. Co-occurrence network of keywords from 2010 to 2019.


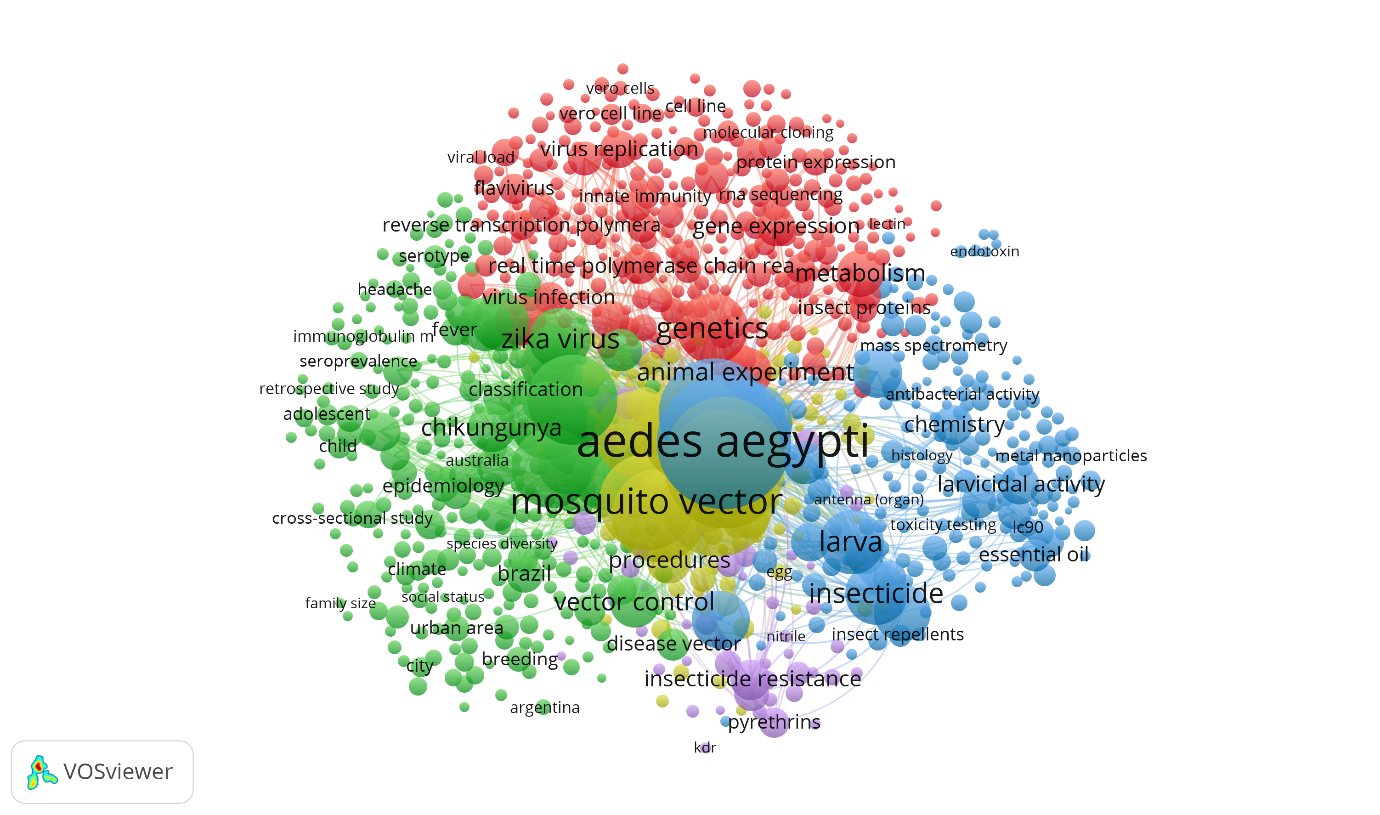


Figure S4. Co-occurrence network of keywords from 2020 to 2023.


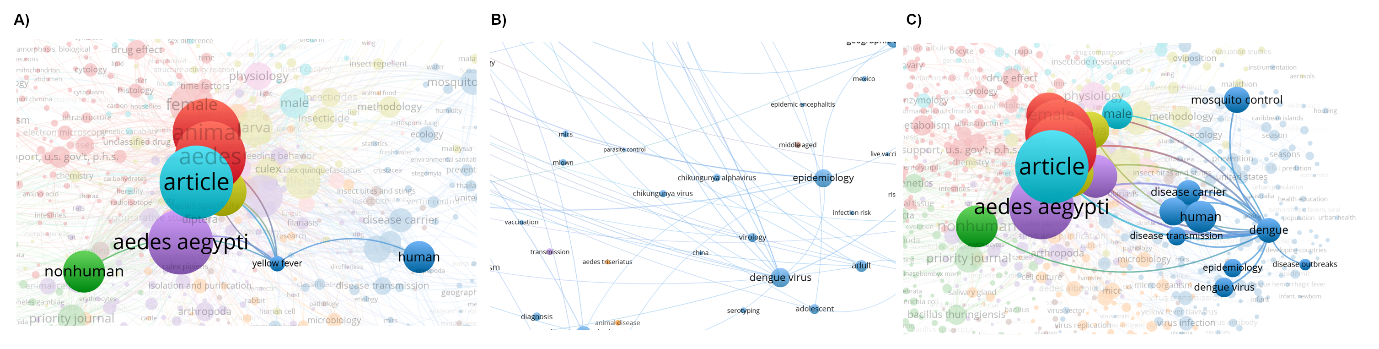


Figure S5. Co-occurrence network of keywords from 1927 to 1999 for (a) Yellow Fever; (b) Chikungunya; (c) Dengue.


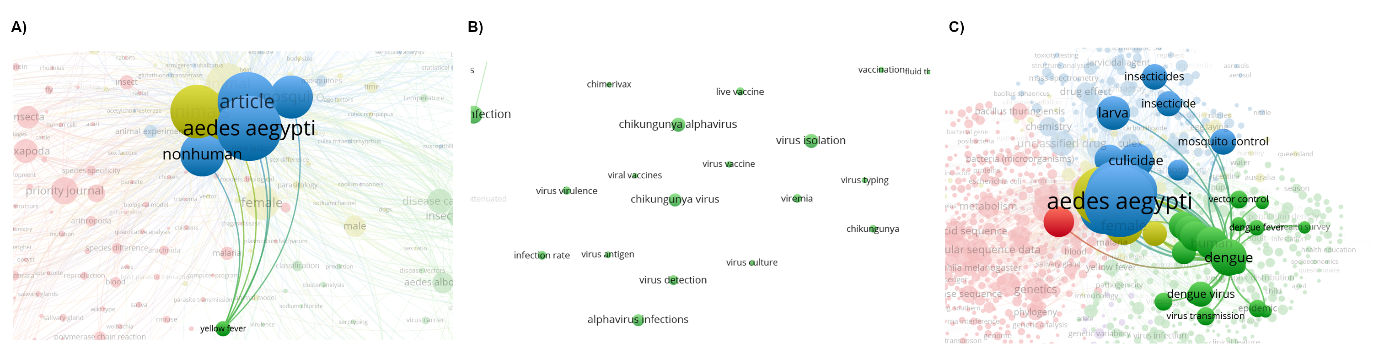


Figure S6. Co-occurrence network of keywords from 2000 to 2009 for (a) Yellow Fever; (b) Chikungunya; (c) Dengue.


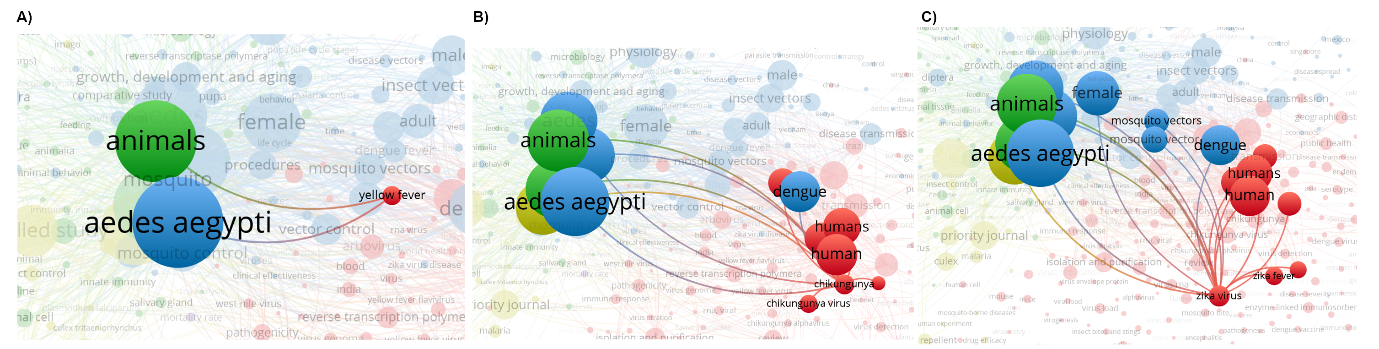


Figure S7. Co-occurrence network of keywords from 2010 to 2019 for (a) Yellow Fever; (b) Dengue and Chikungunya; (c) Dengue and Zika.


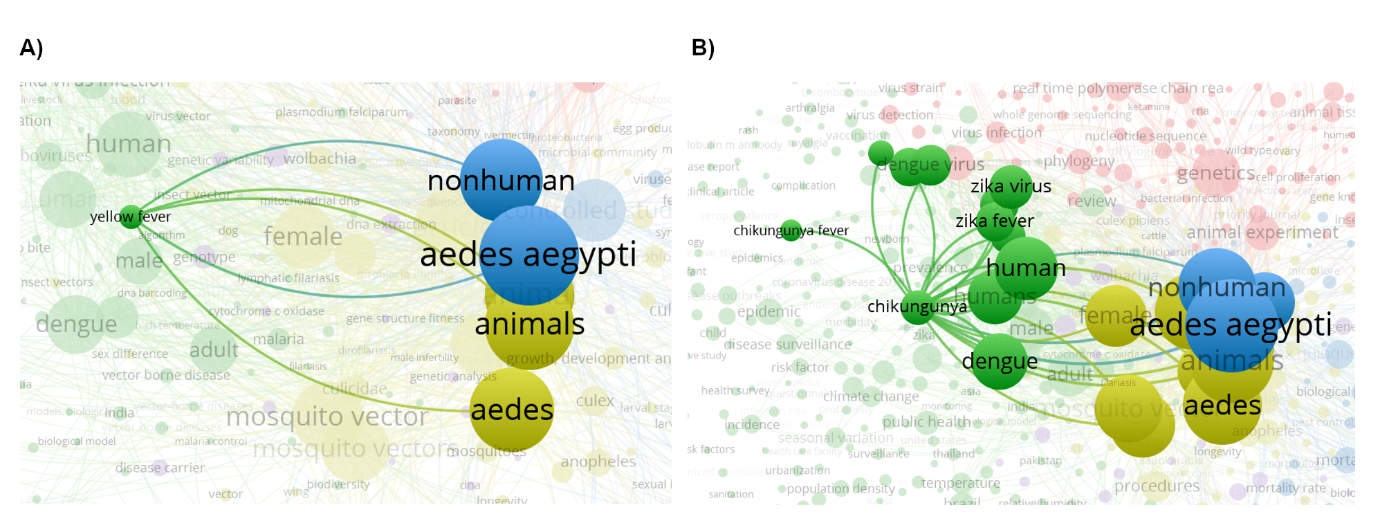


Figure S8. Co-occurrence network of keywords from 2020 to 2023 for (a) Yellow Fever; (b) Chikungunya, Dengue and Zika.
